# An Indirect Cue of Predation Risk Counteracts Female Preference for Conspecifics in a Naturally Hybridizing Fish *Xiphophorus birchmanni*

**DOI:** 10.1371/journal.pone.0034802

**Published:** 2012-04-18

**Authors:** Pamela M. Willis, Gil G. Rosenthal, Michael J. Ryan

**Affiliations:** 1 Section of Integrative Biology, University of Texas at Austin, Austin, Texas, United States of America; 2 Department of Biology, Texas A & M University, College Station, Texas, United States of America; 3 Centro de Investigación Científica de las Huastecas “Aguazarca”, Calnali, Hidalgo, Mexico; Ecole Normale Supérieure de Lyon, France

## Abstract

Mate choice is context dependent, but the importance of current context to interspecific mating and hybridization is largely unexplored. An important influence on mate choice is predation risk. We investigated how variation in an indirect cue of predation risk, distance to shelter, influences mate choice in the swordtail *Xiphophorus birchmanni*, a species which sometimes hybridizes with *X. malinche* in the wild. We conducted mate choice experiments to determine whether females attend to the distance to shelter and whether this cue of predation risk can counteract female preference for conspecifics. Females were sensitive to shelter distance independent of male presence. When conspecific and heterospecific *X. malinche* males were in equally risky habitats (i.e., equally distant from shelter), females associated primarily with conspecifics, suggesting an innate preference for conspecifics. However, when heterospecific males were in less risky habitat (i.e., closer to shelter) than conspecific males, females no longer exhibited a preference, suggesting that females calibrate their mate choices in response to predation risk. Our findings illustrate the potential for hybridization to arise, not necessarily through reproductive “mistakes”, but as one of many potential outcomes of a context-dependent mate choice strategy.

## Introduction

Mate choice is influenced by multiple factors, including mate preference and the current context in which mate choice decisions are made [1], [2]. Internal and environmental conditions influence the costs and benefits individuals accrue from their choice of mate. As a result, mate choice often varies according to environmental factors such as the level of predation risk [3], [4] or the density of potential mates [5], [6], and attributes of the choosers themselves such as age, condition, or reproductive state [7]–[9]. For example, female túngara frogs become less choosy about potential mates as the time remaining for successful reproduction declines [10]. Factors that alter mate choice can in turn affect the strength of sexual selection acting on, or the targets of sexual selection in, the opposite sex. Context-dependent variation in mate choice is therefore important because it may influence evolution by sexual selection; for example, the evolution of novel male traits or the ease of speciation [1], [11]–[12].

While the context-dependence of mate choice within species is well-recognized, less consideration has been given to the possibility that mate choice between species is similarly influenced by current internal and environmental conditions and can result in hybridization. There is evidence, however, that changing conditions can, by altering or constraining mate choice, make hybridization more likely [13]–[17] For example, in hybridizing populations of grebes (*Aechmophorus occidentalis* and *A. clarkii*), males increasingly pursue heterospecific females as conspecific partners become scarce over the breeding season [14]. Understanding the extent to which context-dependent mate choice can promote or inhibit hybridization is important because of its potential to affect the origin, loss, and fate of evolutionary lineages through interspecific gene flow (see [18]–[20]).

A prominent influence on mate choice is predation risk [1]. For many species, mate sampling increases the risk of being detected by predators (e.g., [21]) and individuals often become less choosy when predation risk is high (e.g., [4], [22]). For example, female sand gobies (*Pomatoschistus minutus*) prefer large colorful males, but become indiscriminate around predators [3]. Predation risk might similarly decrease choosiness in potentially hybridizing individuals and thereby increase the chance of hybridization, although this hypothesis has not been tested experimentally.

Many animals rely heavily on indirect cues, such as the distance to cover or level of illumination, to assess the risk of predation [23], [24], and perceived predation risk increases with distance to cover in many species, including fish (e.g., [25], [26]). Here we investigate whether variation in the distance to shelter influences female mate choice in the swordtail fish *Xiphophorus birchmanni*. Hybridization and introgression occur between this species and its congener, *X. malinche*, in several tributaries of the Río Pánuco basin in Hidalgo, Mexico [27], [28]. First generation hybrids occur at very low frequencies, with a greater preponderance of backcross individuals and later generation hybrids [28]. The two species inhabit shallow rocky streams subject to seasonal flooding and drought [29], and likely experience considerable variation in predation pressure, to which swordtails attend [30], [31]. Males of each species differ morphologically in several ways [27], [29]. Previous studies have shown that female *X. birchmanni* typically prefer the cues of conspecific males over those of *X. malinche* [32], [33], but that their choice of mate can vary with environmental conditions [32], [34]. *Xiphophorus birchmanni* is therefore an excellent model for investigating the effects of predation risk on female choice. We experimentally tested female sensitivity to predation risk, and whether it influences their choice of conspecifics over heterospecifics.

## Methods

### Ethics Statement

All research was conducted in compliance with the Guide for the Care and Use of Laboratory Animals. This study was approved by the Institutional Animal Care and Use Committee at The University of Texas, protocol number 07012201. All efforts were made to maximize animal welfare.

### Fish Collection and Experimental Design

We collected *X. birchmanni* from Garces (20°56′24″N, 98°16′54″W), and *X. malinche* from Chicayotla (20°55′26″N, 98°34′35″W) [28], in 2008 and 2009. Subjects and stimuli, all sexually-mature, were either wild-caught or first-generation descendents. Females were isolated from males for at least two weeks before testing.

Water was conditioned with Prime (Seachem Laboratories Inc, Georgia, USA) and carbon-filtered for ≥24 hrs before use. Two filtered 500 W halogen lamps provided downwelling irradiance (UV and visible) comparable to that of natural *Xiphophorus* habitat, following [35]. The sides of the test aquarium (76×30×30 cm) were lined with Teflon and overlain with filter gels, providing diffused horizontal irradiance [35].

**Figure 1:**
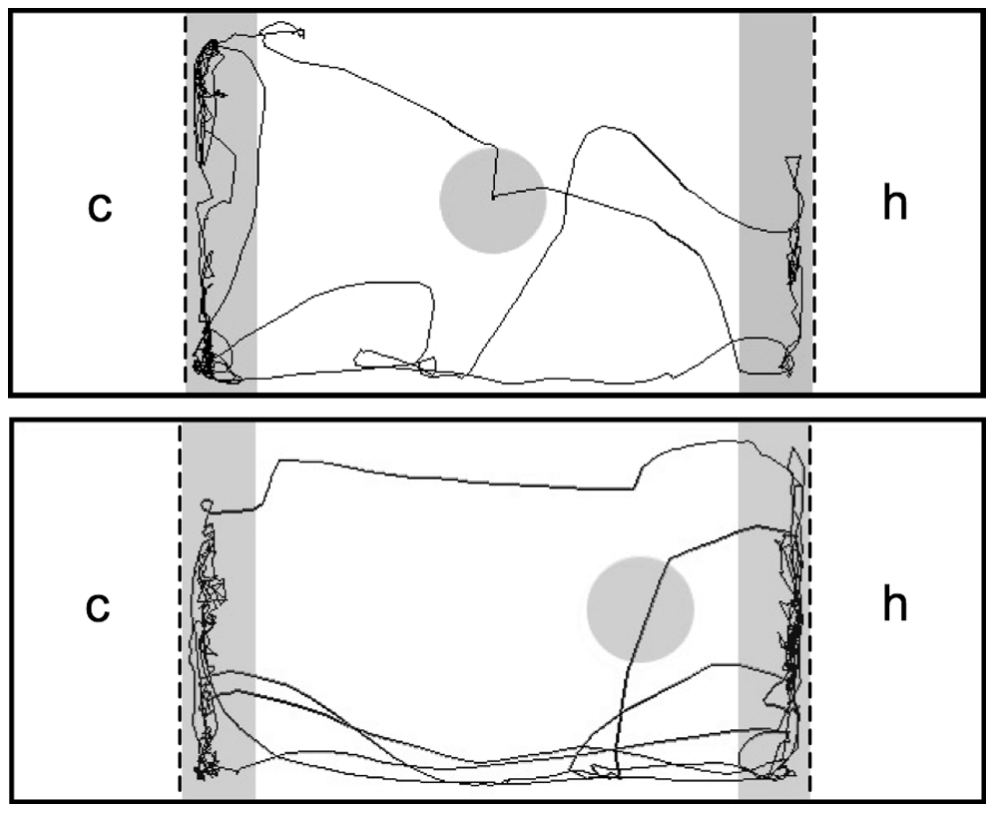
Experimental tank design. Shaded regions represent shelter (circles) and zones (rectangles) in which association time was recorded. Upper panel = shelter equidistant from either male compartment. Lower panel = shelter closer to the heterospecific compartment. c = conspecific male compartment, h = heterospecific male compartment. Drawn to scale. Representative paths of a subject female over two consecutive trials are shown.

Fifteen females were individually offered the choice between conspecific and heterospecific (*X. malinche*) males, presented at opposite ends of the test tank behind clear, UV-transmittant, porous barriers (Fig. 1). The barriers prevent physical interactions between the sexes, while allowing female access to male visual and olfactory cues, both important in *X. birchmanni* mate choice [32], [33]. Males were presented in randomly-assigned groups of three per side to allow females access to within-species phenotypic variation and to reduce male stress (as can occur when males are presented individually, PMW pers. obs.); this design also approaches the setting in the wild, where swordtails occur in large social groups and males court in the presence of other males [36]. Each pair of male groups served as stimuli for three to five females. One *X. malinche* male died during the experiment, and was replaced by a similarly-sized male.

In many species, perceived predation risk increases with distance to cover (e.g., [25], [26]). Swordtails are reluctant to venture far from cover in both the wild [29] and in the lab, and seek shelter immediately when startled (PMW, pers. obs.). We used distance to shelter as an indirect cue of predation risk. Females spontaneously took cover underneath a central shelter (a 7.5 cm diameter sponge filter) upon introduction to the tank. Emergence from beneath the shelter, followed by visits to both sides of the tank, marked the onset of a 3 min acclimation period. We then conducted two consecutive, 5 min trials: one with the shelter equidistant from either male compartment, and one with it closer to the heterospecific side (Fig. 1). Trial order was alternated between females. To minimize disturbance to the fish between trials, the shelter was moved remotely by overhead pulley very slowly over a ∼30 sec period, without removing the fish from the tank. If the first trial was the one with the shelter equidistant from either side, we moved the shelter halfway to the offset position, and then returned it to the center, so as to introduce a comparable amount of shelter movement over the two trials, regardless of trial order. Trials in which the female either hid or was inactive for over half the trial were declared void. As a measure of mate choice we recorded association time with each male stimulus, which predicts mate choice and reproductive success in *Xiphophorus* (e.g., [37]–[39]). We collected data using the automated EthoVision XT video tracking system (version 5.0, Noldus Information Technology, Wageningen, Netherlands). Tanks were emptied, rinsed, and dried between females.

The shelter was visible to males as well as females, allowing for the possibility that males might (also) respond to changes in shelter position. If so, any treatment effect could reflect female responses to changes in male behavior, rather than the distance to shelter. We controlled for potential male-shelter interactions in two ways. First, we recorded two uncorrelated (Pearson *r* = 0.04) measures of male behavior: the number of aggressive events among males within the group (bite attempts and lateral displays, [40]) and overall group activity (total time during which at least one male is actively moving). This allowed us to account for any variation in female behavior arising due to changes in these (or other correlated) male behaviors (see ‘Statistical analyses’). Second, we repeated the experiment without males. This allowed us to observe the influence of shelter position alone on female behavior.

### Statistical Analyses

We used linear mixed models to examine the influence of shelter distance, male species (or side, for trials with no male stimuli), and their interaction on female association time. In the presence of a significant interaction, we performed nested contrasts to evaluate the difference in association time between male species (or side) within each treatment. We used a maximum likelihood protocol implemented by the *lme4* package of R. Female ID and male pair group were treated as random effects to account for the non-independence of within-subject and within-male-group measures. Treatment order was included as a covariate. For trials including male stimuli, male aggression and overall activity were also included as covariates. We used the second-order Akaike Information Criterion (AICc) for model selection [41], using the *MuMIn* package in R. As no competing models ranked highly (i.e. delta AICc<2), model averaging was unnecessary [41]. Model residuals were examined to ascertain assumptions of normality and homogeneity of variances were met.

Conventional significance testing of fixed effects in mixed models is a contentious issue, primarily because it is not clear how to calculate the appropriate degrees of freedom [42]. As null hypothesis testing is a familiar paradigm for many biologists, we used Markov chain Monte Carlo sampling (10000 samples) of the posterior distribution of the parameters to generate 95% posterior density credibility intervals and *p*-values [42] using the *pvals.fnc* function in the *languageR* library of R.

## Results

**Figure 2:**
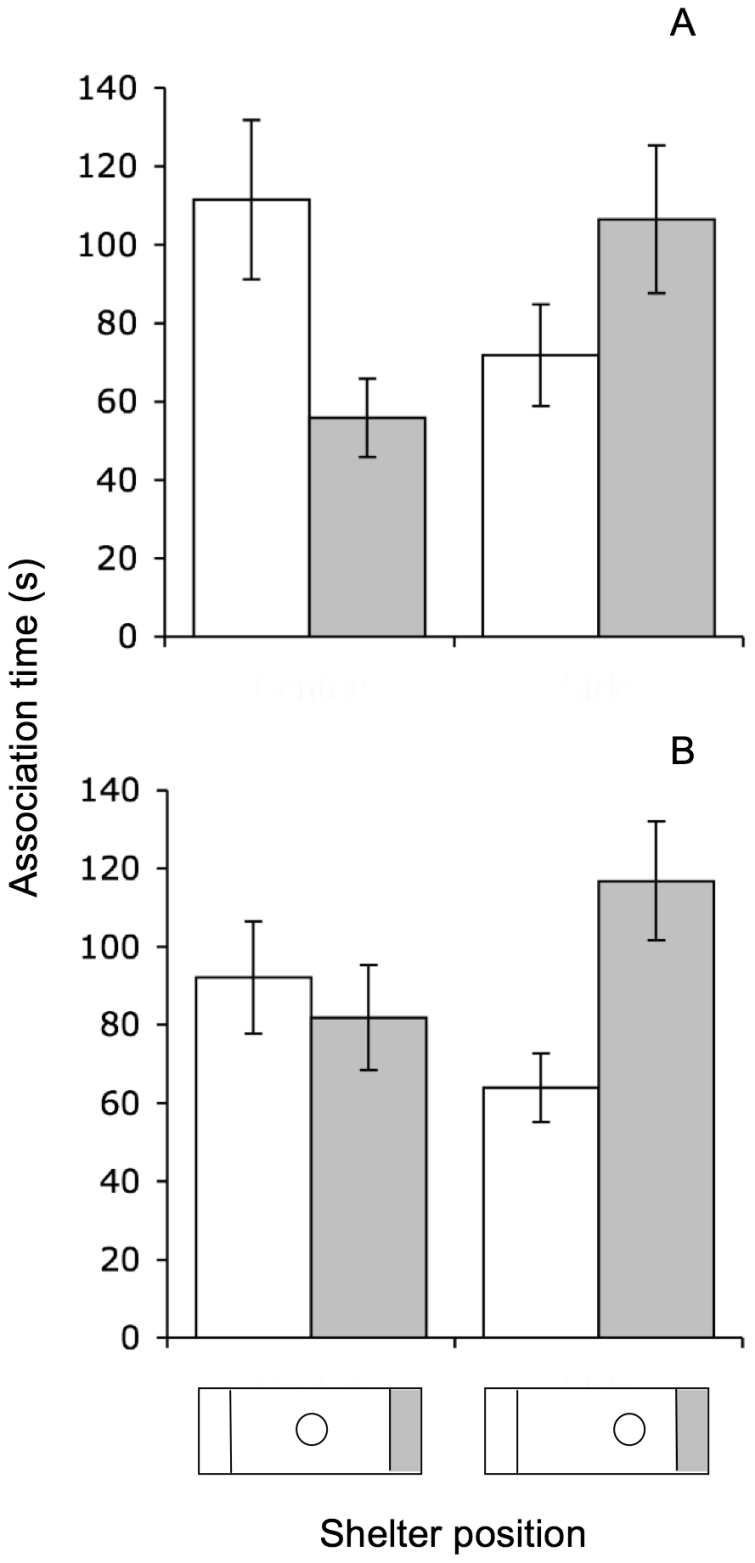
Female time in association zones as a function of male species and shelter position. Female time spent in either zone in the A) presence or B) absence of male stimuli, with the shelter either centered or offset within the tank. In A), white bars = conspecific male side, gray bars = heterospecific male side. In B), bar color designates opposite sides of the tank.

Females preferentially associated with conspecific males when the shelter was equidistant from either species (nested contrast, P_MCMC_ = 0.018; Figure 2A). When the shelter was closer to the heterospecific side, females no longer exhibited a preference (nested contrast, P_MCMC_ = 0.123; linear mixed model, significant male-species-by-shelter-position interaction; Table 1, Figure 2A). A significant effect of male species was also detected, with females associating more with conspecifics overall (Table 1). The two measures of male behavior, aggression and activity, decreased model fit considerably (ΔAIC = 7.249), and were not retained in the final model. No other significant effects were detected (Table 1).

**Table 1:** Best linear mixed models of effects on female association time, in the presence or absence of male stimuli.

Experiment	Parameter	Coefficient estimate	HPD_lower_	HPD_upper_	P_MCMC_
Males present	Male species	−55.67	−102.37	−13.73	0.015
	Shelter position	−39.67	−85.26	2.79	0.073
	Treatment order	14.97	−17.86	47.75	0.372
	Male species * Shelter position	90.33	28.44	153.45	0.006
Males absent	Side	−10.21	−48.76	28.83	0.599
	Shelter position	−28.43	−65.40	9.82	0.138
	Treatment order	6.41	−23.14	35.39	0.677
	Side * Shelter position	63.12	10.26	116.46	0.020

Effect sizes (coefficients) with Bayesian 95% credibility intervals (HPD_lower_, HPD_upper_) and P-values.

In the absence of males, and with the shelter equidistant from either side, females spent their time equally on either side (nested contrast, P_MCMC_ = 0.225; Figure 2B). With the shelter offset, however, females spent more time on the side nearest the shelter (nested contrast, P_MCMC_ = 0.005; Figure 2B), resulting in a significant side-by-shelter-position interaction (Table 1). No other significant effects were detected (Table 1).

## Discussion

In many species, searching for and choosing among potential mates can conflict with predator avoidance (e.g., [21]), causing individuals to adjust their mate choices to the level of risk [3]–[4], [22]. We have shown that, when conspecific and heterospecific males are equally distant from shelter, female *X. birchmanni* prefer conspecifics. Such conspecific preferences are widespread in many taxa, and are important in limiting gene flow between many closely-related species in sympatry [43]–[45]. However, we have also shown that female *X. birchmanni* are sensitive to the perceived risk of predation, and adjust their mate choices accordingly. These findings provide the first experimental evidence that predation risk can override preferences for conspecifics among (actually or potentially) hybridizing species. Our results are significant because animal hybridization can be an important source of evolutionary change [18]–[20]. Predation risk may therefore be important, not only in shaping the form and strength of sexual selection within populations (e.g., [46]), but for introducing genetic novelty between them.

Mate sampling under threat of predation likely occurs In wild *Xiphophorus* [30], [47]. Cichlid fishes co-occur with all northerm swordtail species and are likely important predators [30], [48], as are birds (GGR, unpub. dat.). Other potential predators of *X. birchmanni* include the Mexican tetra (*Astyanax mexicanus*), eleotrid fishes and snakes [47], [49]. Seasonal flooding and drought likely expose individuals to variation in predation risk. For example, drought conditions frequently isolate *Xiphophorus* fishes in small pools (e.g., [50]–[51]), potentially exposing individuals to increased risk of predation, particularly by birds. Conditions of elevated predation risk may constrain female mate choice and increase the likelihood of hybridization.

Although many other species of *Xiphophorus* live in sympatry with others and can hybridize in the lab, hybridization has ony been reported for a few species pairs, and in all cases appears rare (reviewed in [52]). In *X. birchmanni*, both organic pollution [32] and encounter rates with conspecific males [34] reduce female discrimination against *X. malinche* males, in addition to predation risk as reported here. Predation risk may therefore facilitate hybridization in the wild; however, it may be that a variety of conditions are necessary to overcome the strong prezygotic isolation observed in other sympatric species pairs.

Female preference for the sword is common in many *Xiphophorus* species, and this preference is considered ancestral [53]. However, this male ornament is also attractive to predators [47], and predation on sworded males can eliminate female preference for the sword [31]. Swords are both absent in *X. birchmanni* males and unattractive to female conspecifics [29], [33], and the trait has failed to spread across hybrid zones [27]. In the present study, predation risk reduced female discrimination against (sworded) heterospecifics, suggesting that the relationships among female preference, sword presence and predation risk in this species may be complex. Futures studies of the role of the sword in the patterns reported here can help illuminate its complex role in the evolution of *Xiphophorus*.

Given that predation risk influences mate choice in a wide range of species [1], it seems likely that it can alter mate choice in other hybridizing taxa. For example, Enos Lake stickleback species underwent extensive hybridization and subsequent species collapse following the introduction of an omnivorous crayfish [54]–[55], the presence of which inhibits reproductive behavior in one of the parental species [56]; however, the exact mechanisms of this collapse are unknown. Identifying the consequences of hybridization is important for understanding the selective forces affecting reproductive isolation and speciation, and a well-developed literature addresses this topic (e.g., [43], [57]–[59]); however, less often addressed are the behavioral causes of hybridization, which are important for the same reason. Our findings suggest that, in some cases, hybridization may arise as just one of the many potential outcomes of a context-dependent mate choice strategy. Studying the behavioral causes from this perspective may broaden our understanding of the processes reducing or increasing diversity.
